# Acute cardiogenic shock in a girl with systemic lupus erythematosus

**DOI:** 10.4103/0972-5229.76087

**Published:** 2010

**Authors:** Mohan Gurjar, Sanjay Singhal, Banani Poddar, Arvind K Baronia, Afzal Azim

**Affiliations:** **From:** Department of Critical Care Medicine, Sanjay Gandhi Post Graduate Institute of Medical Sciences, Lucknow-226 014, UP, India

**Keywords:** Fulminant myocarditis, steroid, systemic lupus erythematosus

## Abstract

Cardiac involvement is one of the main complications substantially contributing to the morbidity and mortality of patients suffering from systemic lupus erythematous (SLE). However, clinically important myocarditis is an unusual feature in SLE. We describe the clinical characteristics, management, and outcome of a 15-year young girl with SLE who developed severe left ventricular dysfunction.

## Introduction

Systemic lupus erythematosus (SLE) is an autoimmune disease that affects all organ systems including heart. Cardiac involvement is frequent and pericarditis is considered as one of the diagnostic criteria of SLE. Cardiac manifestations could be easily recognized by echocardiography and other noninvasive tests. Although myocarditis is not considered in the standard diagnostic criteria, but it is not an uncommon feature in SLE; and its clinical presentation ranges from asymptomatic patients with self-limited disease to life-threatening heart failure.[1] Here we are reporting a rare case of fulminant myocarditis manifesting as severe congestive heart failure with successful outcome in a young girl with SLE.

## Case Report

A 13-year-old girl presented to the immunology department with the complaint of fever, malar rash, joint pain, diffuse abdominal pain, and history of photosensitivity. Autoimmune profiles revealed hypocomplementemia with C3 of 48.7 mg/dl (normal, 60-120 mg/dl), C4 of 15.9 mg/dl (normal, 16-38 mg/dl), antinuclear antibody titer of more than 4+ with positive coarse speckled at 1:80 and positivity for anti-double-stranded DNA antibody. Other laboratory investigations revealed hemoglobin 6.8 g/dl, total leukocyte count 2800/μl (neutrophil 74%, lymphocyte 25% and eosinophil 1%), platelet count 51000/μl, and routine blood smear picture was suggestive of microcytic hypochromic anemia. So, she was diagnosed as a case of SLE as per revised criteria of American Rheumatism Association for the diagnosis of SLE; and injection hydrocortisone 50 mg intravenous every 6 h started.

**Figure 1:**
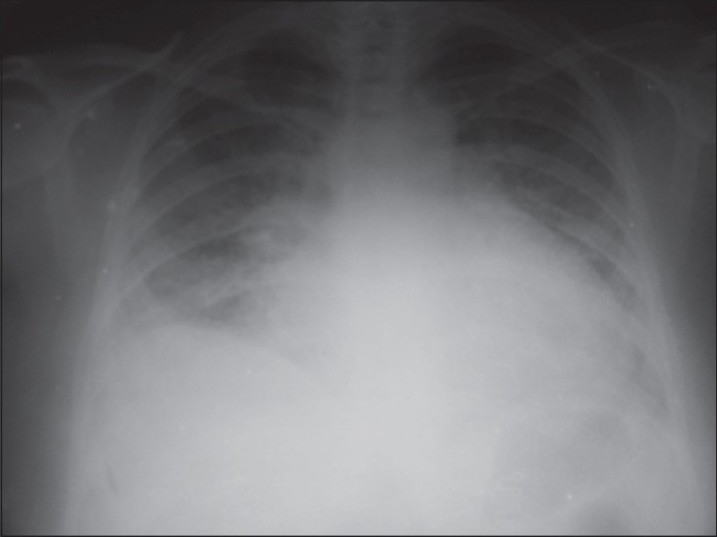
Chest X-ray at ICU admission

On fourth day of admission she was transferred to the critical care unit with respiratory distress, hypotension, tachycardia, and fall in urine output. Electrocardiogram revealed sinus tachycardia with T wave inversion. The chest x-ray showed bilateral diffuse infiltrates in middle and lower lung fields with enlarged cardiac silhouette [Figure 1]. Echocardiography revealed normal left ventricular (LV) size but global hypokinesia with ejection fraction (EF) of only 15%, mild pulmonary artery hypertension, mild pericardial effusion and mild mitral regurgitation.

**Figure 2:**
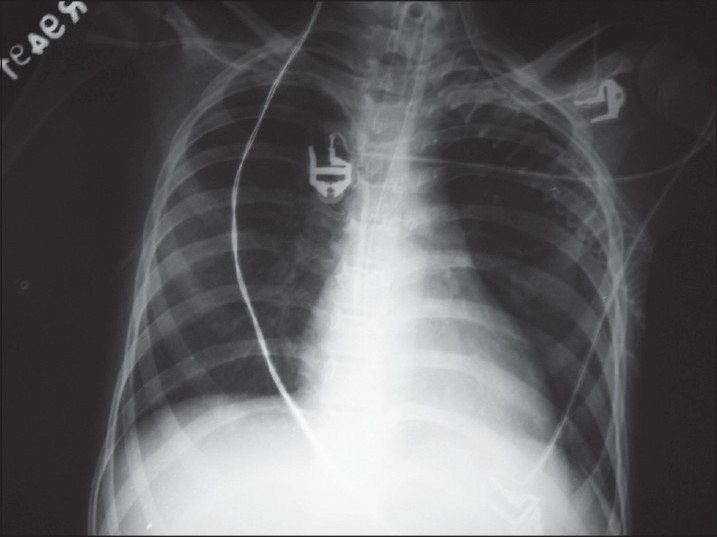
Chest X-ray at time of ICU discharge

In the view of life-threatening myocarditis that leads to cardiogenic shock and pulmonary edema; she required invasive mechanical ventilation as well as vasopressor (norepinephrine) and inotropic (dobutamine) support. On clinical basis, the case was diagnosed as lupus-induced fulminant myocarditis, though we have not done biopsy to confirm and differentiate between viral or lupus-induced myocartitis. Pulse methylprednisolone was given for 5 days. Echocardiography done 4 days later revealed that EF improved from 15% to 30%. She was then started on oral prednisolone at the dose of 1 mg/kg. After stabilizing of hemodynamics, ACE inhibitor and diuretic were started. She was due for weaning but she developed ventilator-associated pneumonia with fever and cultures positive for *Klebsiella pneumoniae.* Sensitivity-based antibiotics were started and she improved clinically as well as rediologically [Figure 2]. At the end of the sixth week of ICU stay she was successfully weaned off from the ventilator. At the time of discharge from ICU, her echocardiography showed marked improvement in LV function, i.e. normal in size, contractility, and EF.

## Discussion

SLE is a multi-system, chronic inflammatory disease affecting all organ systems including all components of heart. Cardiovascular disease is the third major cause of mortality involving 25% of patients with SLE.[2] In SLE, coronary artery disease is the leading cardiovascular manifestation followed by pericarditis, however; myocarditis, endocarditis and valvular disease, conduction abnormality, pulmonary or peripheral arterial hypertension may also occur.[2]

In majority of the SLE patients, myocarditis is asymptomatic. The clinical detection of myocarditis ranges from 3% to 15%, but it appears to be much more common in autopsy studies, suggesting the largely sub-clinical nature of lupus-associated myocarditis.[1,3] Signs and symptoms are similar to those of myocarditis due to other causes [Table 1] like dyspnea, tachycardia, and arrhythmia; and it may progress to ventricular dysfunction, dilated cardiomyopathy, and heart failure. Myocarditis with severe left ventricular (LV) dysfunction has been reported in few adult patients with SLE.[4] On literature search, we found only two cases of clinically apparent lupus myocarditis in children.[5]

**Table I:** Causes of myocarditis

**Infection**
*Viral:* Coxsackie (AandB), echovirus, influenza, cytomegalovirus, rubella virus, measles virus, mumps virus, arbovirus, adenovirus, poliovirus, parvovirus, herpes simplex, respiratory syncytial virus, Epstein-Barr virus, hepatitis, varicella zoster, human immunodeficiency virus
*Bacterial:* streptococcal, staphylococcal, meningococcal, salmonella, diphtheria, tuberculosis
*Fungi: Aspergillus, Candida*
*Parasitic:* trypnosoma, toxoplasma, malaria
*Richettsial*
**Non-infectious**
Giant cell
Eosinophilic
Polyarteritis nodosa
Systemic lupus erythematosus
Ulcerative colitis
Chemical or drug hypersensitivity
Radiation
Trauma
Idiopathic

There are nonspecific findings on ECG, and cardiac enzymes may be normal. Echocardiographic studies cannot definitely diagnose myocarditis, but global hypokinesia, in the absence of other known causes, is strongly suggestive; and the segmental areas of hypokinesia can also be indicative of disease. Though, clinical diagnosis of acute fulminate myocarditis can be made by the presence of severe and acute heart failure, left ventricle dysfunction on echocardiography and no history of cardiomyopathy; but the endomyocardial biopsy still remain gold standard to diagnose as well as to differentiate with other causes of myocarditis. However, the endomyocardial biopsy is an invasive procedure and subject to sampling error. Therefore, the diagnosis of myocarditis in SLE depends largely on the clinical suspicion and echocardiographic findings. Recently, magnetic resonance imaging (MRI) is used for diagnosing myocarditis in SLE, and it appear promising in early diagnosis as being noninvasive.

Current treatment strategies are based on clinical experience rather than randomized trials. Treatment of lupus myocarditis should include an angiotensin-converting enzyme inhibitor and corticosteroids.[6] Few case report of severe form of lupus myocarditis showed promising results for the use intravenous pulse corticosteroid (e.g., methyl prednisolone of 1.0 g/day for 5-7 days) followed by high oral doses (e.g., prednisolone; 1 mg/kg/day). Other therapies like immunosuppressive agents (azathioprine[7] or cyclophosphamide[8]) and intravenous immunoglobulin[9] (IVIG) has shown beneficial effect in lupus myocarditis. Efficacy of the therapy can be assessed by serial echocardiographic studies or endomyocardial biopsies.

In a recent study, survival for the first 5 years in SLE patients with myocarditis and without myocarditis was similar, but after 5 years mortality almost doubles in the myocarditis group as compared to patients without the myocarditis group (18.9% vs. 8.4%).[10]

This case highlights that the presence of lupus myocarditis merits an urgent intervention because of the potentially devastating consequences that might lead to death. Early diagnosis and prompt treatment with high dose steroid may result in good outcome.

## References

[1] Wijetunga M, Rockson S (2002). Myocarditis in systemic lupus erythematosus (review). Am J Med.

[2] Szekanecz Z, Shoenfeld Y (2006). Lupus and cardiovascular disease: The facts. Lupus.

[3] Doria A, Iaccarino L, Sarzi-Puttini P, Atzeni F, Turriel M, Petri M (2005). Cardiovascular involvement in systemic lupus erythematous. Lupus.

[4] Woo SI, Hwang GS, Kang SJ, Park JS, Park SJ, Lee YS (2009). Lupus myocarditis presenting as acute congestive heart failure: A case report. J Korean Med Sci.

[5] Gupta A, Singh S, Minz RW, Radotra BD (2004). Lupus myocarditis in children. Ann Rheum Dis.

[6] Frustaci A, Gentiloni N, Caldarulo M (1996). Acute myocarditis and left ventricular aneurysm as presentations of systemic lupus erythematous. Chest.

[7] Naarendorp M, Kerr LD, Khan AS, Omstein MH (1999). Dramatic improvement of left ventricular function after cytotoxic therapy in lupus patient with acute cardiomyopathy: Report of 6 cases. J Rheumatol.

[8] Chan YK, Li EK, Tam LS, Chow LT, Ng HK (2003). Intravenous cyclophosphamide improves cardiac dysfunction in lupus myocarditis. Scan J Rheumatol.

[9] Sherer Y, Levy Y, Shoenfeld Y (1999). Marked improvement of severe cardiac dysfunction after one course of intravenous immunoglobulin in a patient with systemic lupus erythematosus. Clin Rheumatol.

[10] Apte M, McGwin G, Vilá LM, Kaslow RA, Alarcón GS, Reveille JD (2008). Associated factors and impact of myocarditis in patients with SLE from LUMINA, a multiethnic US cohort (LV). Rheumatology.

